# Hospitalization Duration for Acute Myocardial Infarction: A Temporal Analysis of 18-Year United States Data

**DOI:** 10.3390/medicina58121846

**Published:** 2022-12-15

**Authors:** Anusha G. Bhat, Mandeep Singh, Sri Harsha Patlolla, Peter Matthew Belford, David X. Zhao, Saraschandra Vallabhajosyula

**Affiliations:** 1 Division of Cardiovascular Medicine, Department of Medicine, University of Maryland School of Medicine, Baltimore, MD 21201, USA; 2 Department of Cardiovascular Medicine, Mayo Clinic, Rochester, MN 55901, USA; 3 Department of Cardiovascular Surgery, Mayo Clinic, Rochester, MN 55901, USA; 4 Section of Cardiovascular Medicine, Department of Medicine, Wake Forest University School of Medicine, Winston Salem, NC 27157, USA

**Keywords:** length of stay, acute myocardial infarction, hospital stay, resource utilization, outcomes

## Abstract

*Background and objectives*: Primary percutaneous coronary intervention (PCI)-related outcomes in acute myocardial infarction (AMI) have improved over time, but there are limited data on the length of stay (LOS) in relation to in-hospital mortality. *Materials and Methods*: A retrospective cohort of adult AMI admissions was identified from the National Inpatient Sample (2000–2017) and stratified into short (≤3 days) and long (>3 days) LOS. Outcomes of interest included temporal trends in LOS and associated in-hospital mortality, further sub-stratified based on demographics and comorbidities. *Results*: A total 11,622,528 admissions with AMI were identified, with a median LOS of 3 (interquartile range [IQR] 2–6) days with 49.9% short and 47.3% long LOS, respectively. In 2017, compared to 2000, temporal trends in LOS declined in all AMI, with marginal increases in LOS >3 days and decreases for ≤3 days (median 2 [IQR 1–3]) vs. long LOS (median 6 [IQR 5–9]). Patients with long LOS had lower rates of coronary angiography and PCI, but higher rates of non-cardiac organ support (respiratory and renal) and use of coronary artery bypass grafting. Unadjusted in-hospital mortality declined over time. Short LOS had comparable mortality to long LOS (51.3% vs. 48.6%) (*p* = 0.13); however, adjusted in-hospital mortality was higher in LOS >3 days when compared to LOS ≤ 3 days (adjusted OR 3.00, 95% CI 2.98–3.02, *p* < 0.001), with higher hospitalization (*p* < 0.001) when compared to long LOS. *Conclusions*: Median LOS in AMI, particularly in STEMI, has declined over the last two decades with a consistent trend in subgroup analysis. Longer LOS is associated with higher in-hospital mortality, higher hospitalization costs, and less frequent discharges to home compared to those with shorter LOS.

## 1. Introduction

Despite an increase in the risk and complexity, the success and safety of primary percutaneous coronary intervention (PCI) for acute myocardial infarction (AMI) have continued to improve, underscoring the need to review the determinants of the length of stay (LOS) during index hospitalization that may have significant cost implications [1,2]. The main drivers for prolonged LOS following PCI are post-procedure bleeding, acute stent thrombosis, fatal arrhythmias, or management of associated comorbid conditions. The use of radial access, newer stent designs, and modification of the antiplatelet protocols have reduced the incidence of stent thrombosis and bleeding complications [3].

The current European guidelines and the consensus decision from the American College of Cardiology recommend dismissal at 48–72 h following ST-segment elevation myocardial infarction (STEMI) of low-risk patients [4,5]. The safety of early dismissal of STEMI patients at 24–72 h has been reported [5]; however, the data are derived from small, older randomized trials or more contemporary single-center observational studies [5]. Additionally, no data are available among patients with non-STEMI (NSTEMI), the most common type of acute myocardial infarction (AMI) in the contemporary era [6]. We, therefore, sought to evaluate LOS in relation to inpatient mortality following STEMI and NSTEMI in the contemporary era from a nationally representative United States population. We also assessed temporal trends of LOS in all AMI and in subgroups based on demographics and comorbidities as other outcomes.

## 2. Material and Methods

The National (Nationwide) Inpatient Sample (NIS) is a database and software partnered by Federal-State-industry and funded by the Agency for Healthcare Research and Quality for the Healthcare Cost and Utilization Project (HCUP). The database is the largest all-payer database of hospital inpatient stays in the United States. It was created to generate national estimates of inpatient usage, access to healthcare, hospital costs, and outcomes. It covers more than 97% of the US population and approximates a 20% stratified sample of all the US hospital discharges [7]. The database extracts deidentified information such as demographics, primary payer, comorbidities, principal diagnosis, up to 29 secondary diagnoses, and procedural diagnoses for each discharge from all the states participating in the HCUP. Since the information available through this database is publicly available and de-identified, Institutional Review Board approval was not necessary. Observations were identified as ‘admissions’ rather than being considered as individual patients. We used validated administrative codes, and we confined the variables to inpatient ones as the HCUP-NIS is limited by a lack of validation of its outpatient information.

### 2.1. Study Population, Variables, and Outcomes

We queried HCUP-NIS data from 1 January 2000 to 31 December 2017 to identify adults (>18 years) with AMI in the primary diagnosis field (International Classification of Diseases 9.0 Clinical Modification [ICD-9CM] 410. x) [8,9,10]. AMI admissions without documented LOS were excluded. Prior studies have determined AMI administrative codes to have high sensitivity (98%) and specificity (91%), positive predictive value (95%), and negative predictive value (97%). Deyo’s modification of the Charlson Comorbidity Index was used to recognize comorbidities [11]. Demographic details such as age, sex, race, in-hospital events, comorbidities, cardiac procedures, and other invasive and non-invasive procedures were obtained using the methodologies previously used by our group (Supplementary Table S1) [8,9,10,12,13,14,15,16,17,18]. Non-cardiac organ failure and multi-organ failure were defined in consistency with our prior work, where administrative codes were used to identify one or more organ systems’ involvement other than cardiovascular failure ^8^.

Due to the skewed nature of the length of stay (LOS) data toward longer LOS in terms of the mean ± standard deviation, it was evaluated using the median (interquartile range [IQR]). Similar to prior data, the LOS was divided into short (≤median LOS) and long (>median LOS) [19]. The primary outcomes of interest were temporal trends of hospitalizations stratified based on “short” (≤3 days) and “long” (>3 days) LOS in AMI. The secondary outcomes of interest were temporal trends in LOS stratified based on sex, presence or absence of cardiogenic shock, cardiac arrest, and in-hospital mortality outcomes. 

### 2.2. Statistical Analysis

National estimates were created using discharge weights provided by the HCUP ^7^. Given the redesigns of NIS data since 2012, weights to produce national estimates were limited to discharge weights. Th HCUP-NIS issued the trend weights for re-weighing samples from 2000 to 2011, which were used to adjust for the 2012 HCUP-NIS redesign [20]. Pearson’s chi-squared tests were used for categorical variables (demographics, in-hospital characteristics, and clinical outcomes of AMI), ANOVA was carried out for comparison of LOS with demographic groups such as race, primary payer, median household income, Charlson Comorbidity Index, etc., and two-sided t-tests were used to compare continuous variables. Temporal trends in LOS stratified based on sex and clinical outcomes were plotted after sub-stratifying for the type of AMI. Univariable analysis was performed to assess trends and outcomes, represented as the odds ratio (OR) with 95% confidence interval (CI). Multivariable logistic regression was performed to analyze trends over time (referred to year 2000), and the ORs with 95% CIs were calculated for each year adjusting for age, sex, race, income status, comorbidities, primary payer, hospital characteristics, acute organ failure, cardiac arrest, cardiogenic shock, coronary angiography, PCI, pulmonary artery catheter use, mechanical circulatory device use, invasive and non-invasive mechanical ventilation, and acute hemodialysis. For the multivariable modeling, regression analysis with purposeful selection of statistically (liberal threshold of *p* < 0.20 in univariate analysis) and clinically relevant variables was conducted. 

Two-tailed *p* < 0.05 was considered statistically significant. Given the large sample size, all *p* values that were statistically significant may not have been clinically significant. All statistical analyses were performed using SPSS version 28.0 (IBM Corp, Armonk, NY, USA). 

## 3. Results

**Figure 1:**
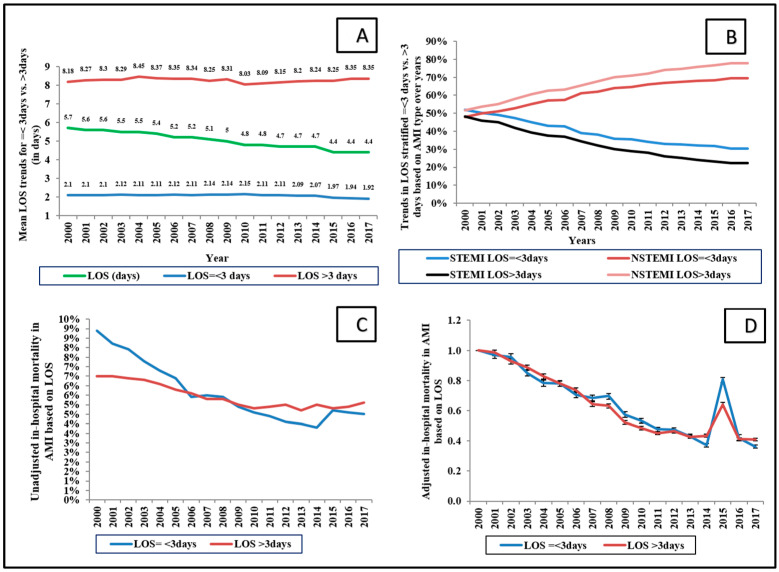
Trends in overall length of stay in acute myocardial infarction. Legend: (**A**) Unadjusted temporal trends in mean length of stay in AMI (*p* < 0.001 for trend over time); (**B**) unadjusted length of stay stratified based on type of AMI and LOS (≤3 days vs. >3 days) in the hospital in AMI; (**C**): unadjusted in-hospital mortality in AMI based on LOS; (**D**): adjusted in-hospital mortality in AMI based on LOS (with 2000 as the referent); *p* < 0.001 for trend over time. Adjusted for age, sex, race, income status, comorbidity, primary payer, hospital region, hospital location, teaching status, hospital bed size, type of MI, cardiogenic shock, cardiac arrest, acute organ failure, atrial fibrillation, atrial flutter, coronary angiography, percutaneous coronary intervention, coronary artery bypass grafting, pulmonary artery catheterization, mechanical circulatory support, invasive and non-invasive mechanical ventilation, and acute hemodialysis (*p* < 0.001 for trend over time). Abbreviations: AMI: acute myocardial infarction, LOS: length of stay.

During the study period between 1 January 2000 and 31 December 2017, we identified 11,622,528 admissions with AMI with a median LOS of 3 (interquartile range: 2–6) days (mean ± standard deviation 5 ± 5.8 days). Of these, 5,810,565 (49.9%) admissions had a short LOS (≤3 days) and 5,499,437 (47.3%) had a prolonged LOS (>3 days); other AMI admissions (2.7%) without documented LOS were excluded. In comparison to those with short LOS, the cohort with long LOS were more often older, female, bearing Medicare insurance, with higher co-morbidities, and admitted with a NSTEMI (*p* < 0.001) (Table 1). Overall, during the 18-year study period, there was a temporal decrease in mean LOS in all AMI, with a marginal decrease in shorter, but an increase in longer LOS (Figure 1A,B).

**Figure 2:**
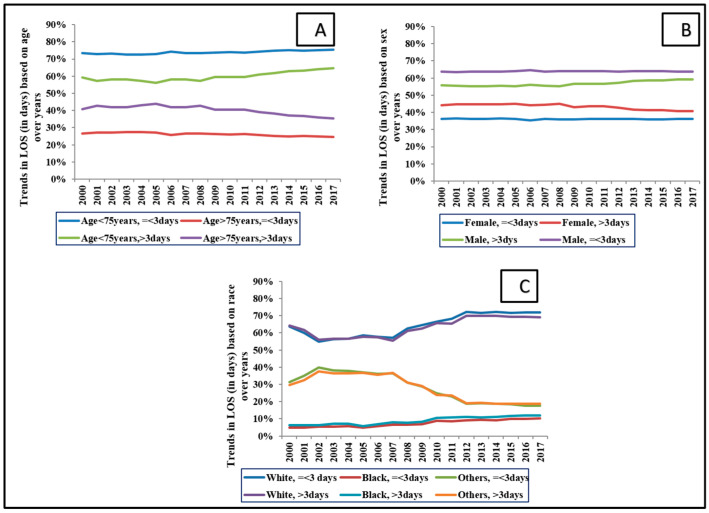
Temporal trends of LOS (in days) based on patient characteristics. Legend: Percentage of AMI admissions with LOS based on age (**A**), sex (**B**), and race (**C**). Abbreviations: AMI: acute myocardial infarction, LOS: length of stay.

LOS in a STEMI population decreased, whereas it increased in a NSTEMI population (Figure 1B). When stratified by patient characteristics, LOS remained steady except for a marginal uptrend in LOS in the white and black race categories (Figure 2). There was a significant difference in short vs. long LOS based on means of demographics: race, primary payer, median income based on zip code, hospital size, location, hospital teaching status, and comorbidity (*p* < 0.001).

**Figure 3:**
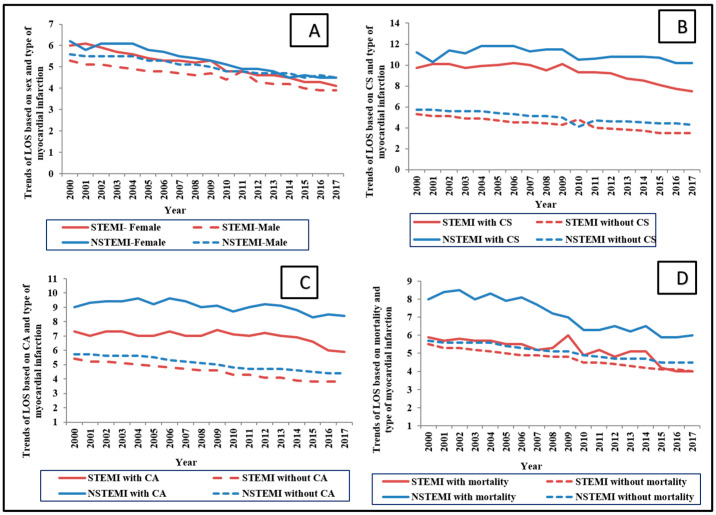
Temporal trends for LOS in AMI based on clinical and co-morbid conditions. Legend: A: Temporal trends LOS based on sex (**A**), presence of CS (**B**), CA (**C**), and mortality (**D**), stratified by type of AMI (*p* < 0.001 for trend over time for all). Abbreviations: AMI: acute myocardial infarction; CA: cardiac arrest; CS: cardiogenic shock; NSTEMI: non-ST-segment elevation myocardial infarction; STEMI: ST-segment elevation myocardial infarction.

The cohort with a more extended stay had a more complicated hospitalization course, as noted by a higher prevalence of cardiogenic shock, cardiac arrest, and non-cardiac organ failure (Table 2). In addition, this cohort had a higher prevalence of ventricular tachycardia/fibrillation, atrial tachyarrhythmia, stroke, and a higher prevalence of in-hospital complications with vascular injury, bleeding, and blood transfusion (Table 2). The cohort with higher LOS had lower coronary angiography and percutaneous coronary intervention (PCI) but higher coronary artery bypass grafting surgery and non-cardiac organ support (Table 2). When LOS was stratified based on the type of AMI, the temporal trends in hospital stay showed a decline independent of sex, presence of cardiogenic shock, cardiac arrest, or mortality (Figure 3A–C). 

Adjusted and unadjusted in-hospital mortality declined over time in both groups (Figure 1C,D). Unadjusted in-hospital mortality was comparable between the two groups (51.3% vs. 48.6%, *p*= 0.13); however, adjusted in-hospital mortality was higher in LOS > 3 days when compared to LOS ≤ 3 days (adjusted OR 3.00, 95% CI 2.98–3.02, *p* < 0.001) (Supplementary Table S2). Additionally, hospitalization costs were higher with longer LOS, and this cohort was discharged more often to skilled nursing facilities or homes with home healthcare (*p* < 0.001) (Table 3). 

## 4. Discussion

In the current study evaluating the impact of LOS on outcomes in AMI, the median LOS was noted to be gradually declining over time. Admissions with longer LOS had higher comorbidities and in-hospital complications including single and multiorgan failure, cardiac arrest, cardiogenic shock, and non-cardiac organ support requirements and received higher rates of surgical as compared to percutaneous revascularization. After adjustment of confounding demographics and comorbidities, longer LOS still had a nearly three times higher risk of in-hospital mortality compared to short LOS. 

An earlier study from the pre-2000 era noted a 50% decline in LOS in 1997–99 (5.9 days) compared to 1986–88 (11.7 days) among a population-based study [21]. Since then, small, randomized trials and a recent meta-analysis have demonstrated the safety of early dismissal among lower-risk patients [22,23]. A recent, single-center study of 600 patients with STEMI confirmed the safety of early dismissal [24]. Efforts toward early dismissal rest on identifying lower-risk patients by utilizing risk models such as ZWOLLE and GRACE and the absence of any bleeding, arrhythmia, or hemodynamic complications following successful PCI. We recently performed a prospective study among patients undergoing PCI (72.8% acute coronary syndrome) and determined that only 2.2% had actionable alarms that included fatal arrhythmias [25]. The median time of onset of arrhythmias was <6 h, underscoring the safety of early-dismissal protocols. The majority of events (bleeding, thrombosis, arrhythmias, and hemodynamic instability) happen within a few hours following primary PCI. Predictors of arrhythmia, bleeding, and hemodynamic instability overlap and can be easily identified following the completion of PCI. The success of PCI has improved to a level where most AMI patients, including STEMI, can be safely discharged following a few hours of observation [1]. Yet, safety concerns, limitations in opportunities for patient education, and lack of strategies to optimize the patient’s risk factor profile remain potential barriers to early dismissal. The current European guidelines still recommend dismissal at 48–72 h following STEMI of low-risk patients. No data are available for such measures among patients with NSTEMI, the most common type of AMI [26]. 

Our study demonstrated overall trends of decrease in LOS, mainly for STEMI whereas the trends increased steeply for patients presenting with NSTEMI. Patients with a higher comorbidity burden and challenging course, including a presentation with shock or cardiac arrest, stayed longer during an index hospital stay. In addition, management of comorbid conditions, the need for urgent CABG, and long-term care placement were additional determinants of LOS and higher mortality. 

Interestingly, we found the unadjusted in-hospital mortality difference between the two LOS groups to be comparable, which changed after adjusting for comorbidities. Prior work from the HCUP-NIS by Jang et al. determined lower 30-day readmission rates and lower costs but at the expense of higher 30-day mortality among patients whose LOS was 1–2 days (HR 1.91; CI, 1.16–3.16) as compared to a LOS of 3 days, underscoring the need to monitor these patients closely following the discharge [27]. In an older study from Seattle area hospitals, the demographic and clinical characteristics explained 6%, and hospital complications, procedure use, and type an additional 27%; however, 29% of the variations in the LOS could not be explained based on the measured variables [28]. This is likely due to unmeasured clinical (frailty, comorbid conditions), administrative, hospital-related, or economic factors that determine the LOS of these patients and underscore the need for uniform systems of care.

### Study Limitations

The HCUP-NIS has used several quality assurance measures to curtail errors, but has not been able to eliminate basic limitations that can impact research designs, statistical analysis, and data interpretation, which we took into account [20]. First, the data are observational and do not reliably identify etiologies for short or long lengths of stay, laboratory findings, or details about the medical therapies used to treat AMI. Second, accurate LOS based on the exact time of admission to discharge is not determinable given the nature of the database. Similarly, given the nature of the database, it is not possible to delineate type-1 from type-2 AMIs, but we included the AMI that primarily caused the admission, which effectively excluded type-2 AMIs since they would have had an alternate primary diagnosis as a trigger. Third, identification of AMI was based on appropriate documentation of the ICD codes. We noted a sharp increase in adjusted mortality trends in Figure 1D in the year 2015, likely due to the transition from ICD-9 to ICD-10 despite controlling for confounding. Lastly, due to reasons mentioned in our methods section, our results are limited to in-hospital outcomes and may not be extrapolated to long-term consequences; this is salient insofar as survivors of AMI hospitalization may have adverse outcomes after discharge from the hospital.

## 5. Conclusions

In conclusion, in this 18-year national study, we found the median LOS to decline steadily, including in subgroups stratified based on sex, cardiogenic shock, cardiac arrest, and mortality outcomes, except in NSTEMI. While unadjusted in-hospital mortality rates in short and long LOS admissions were not different, adjusting for comorbidities increased the mortality in long LOS by threefold. Longer LOS also incurred higher in-hospital complications, requiring higher organ support, and higher hospitalization costs. Much remains unknown about contributors to poor outcomes in NSTEMI and longer LOS, which needs further study across the population with AMI. 

## Figures and Tables

**Table 1:** Baseline characteristics of AMI admissions LOS ≤ 3 days and LOS > 3 days.

Characteristic (N = 11,622,528)		LOS ≤ 3 Days (N = 5,810,565)	LOS > 3 Days (N = 5,499,437)	*p*
**Age (years, mean ± standard deviation)**		64.9 ± 14.2	70.3 ± 13.6	<0.001
**Female (%)**		36.1	43.5	<0.001
**Race (%)**	**White**	64.7	62.5	<0.001
	**Black**	7.5	8.5	
	**Others ^a^**	27.8	29.0	
**Primary payer (%)**	**Medicare**	49.8	65.8	<0.001
	**Medicaid**	6.4	6.0	
	**Private**	34.0	21.4	
	**Others ^b^**	9.8	6.8	
**Quartile of median household income for zip code (%)**	**0–25^th^**	24.0	24.8	<0.001
	**26^th^–50^th^**	27.3	27.0	
	**51^st^–75^th^**	24.8	24.2	
	**75^th^–100^th^**	23.9	24.0	
**Charlson Comorbidity Index (%)**	**0–3**	48.5	25.9	<0.001
	**4–6**	39.2	49.9	
	**≥7**	12.3	24.2	
**Cardiogenic shock (%)**		2.5	6.8	<0.001
**Cardiac arrest (%)**		3.6	5.9	<0.001
**Hypertension (%)**		62.3	62.9	<0.001
**Hyperlipidemia (%)**		52.9	42.4	<0.001
**Heart failure (%)**		4.4	9.8	<0.001
**Hospital teaching** **status and location (%)**	**Rural**	11.9	9.3	<0.001
	**Urban non-teaching**	39.7	39.2	
	**Urban teaching**	48.4	51.5	
**Hospital bed-size (%)**	**Small**	12.4	9.5	<0.001
	**Medium**	26.5	24.2	
	**Large**	61.2	66.3	
**Hospital region (%)**	**Northeast**	17.9	21.5	<0.001
	**Midwest**	23.5	22.1	
	**South**	39.3	41.0	
	**West**	19.3	15.4	

Legend: ^a^ Hispanic, Asian or Pacific Islander, Native American, Others; ^b^ Self-Pay, No Charge, Others. Abbreviations: AMI: acute myocardial infarction; LOS: length of stay.

**Table 2:** In-hospital characteristics of AMI admissions LOS ≤ 3 days and LOS > 3 days.

Characteristic (N = 11,622,528)		LOS ≤ 3 Days (N = 5,810,565)	LOS > 3 Days (N = 5,499,437)	*p*
**AMI type (%)**	**ST-segment elevation AMI**	38.6	34.6	<0.001
	**Non-ST-segment elevation AMI**	61.4	65.4	
**Acute non-cardiac organ failure (%)**	**Overall**	4.0	15.1	<0.001
	**Respiratory**	3.9	13.3	<0.001
	**Hepatic**	0.5	1.5	<0.001
	**Renal**	5.6	18.4	<0.001
	**Hematologic**	1.7	5.9	<0.001
	**Neurologic**	1.5	4.9	<0.001
**In-hospital events (%)**	**Ventricular arrhythmias**	5.9	10.2	<0.001
	**Atrial fibrillation**	10.7	23.4	0.048
	**Systolic heart failure**	4.4	9.8	<0.001
	**Stroke**	0.7	2.9	<0.001
	**Ischemic stroke**	0.6	2.7	<0.001
	**Intracranial hemorrhage**	0.1	0.3	<0.001
	**Acute pulmonary embolism**	0.1	0.6	<0.001
**Cardiac procedures (%)**	**Coronary angiography**	67.7	61.6	<0.001
	**Percutaneous coronary intervention**	51.6	32.6	<0.001
	**Coronary artery bypass grafting**	0.2	19.2	<0.001
	**Fibrinolytics**	2.0	1.5	<0.001
	**Mechanical circulatory support**	1.6	8.0	<0.001
	**Pulmonary artery catheterization**	0.3	2.0	<0.001
**Non-cardiac procedures (%)**	**Invasive mechanical ventilation**	2.8	9.0	<0.001
	**Non-invasive mechanical ventilation**	0.7	2.4	<0.001
	**Acute hemodialysis**	0.1	1.1	<0.001
**Complications (%)**	**Ventricular septal defect**	0.1	0.1	0.56
	**Hemorrhage**	0.3	4.1	<0.001
	**Vascular injury**	0.4	1.3	0.531
	**Blood transfusion**	1.6	12.0	<0.001
**Palliative care consultation (%)**		1.0%	1.4	<0.001
**Do not resuscitate status (%)**		2.3%	2.7	<0.001

Legend: Represented as percentage or mean ± standard deviation. Abbreviations: AMI: acute myocardial infarction; LOS: length of stay.

**Table 3:** Clinical outcomes of AMI admissions with LOS ≤ 3 days and LOS > 3 days.

Characteristic (N = 11,622,528)		LOS ≤ 3 Days (N = 5,810,565)	LOS > 3 Days (N = 5,499,437)	*p*
**In-hospital mortality (%)**		51.3	48.6	0.13
**Hospitalization costs (×1000 United States Dollars)**		39 (14–54)	54 (28–101)	<0.001
**Discharge disposition (%)**	**Home**	73.5	53.2	<0.001
	**Transfer**	16.6	5.3	
	**Skilled nursing facility**	4.5	23.2	
	**Home with home healthcare**	4.1	17.9	
	**Against medical advice**	1.2	0.4	

Legend: Represented as percentage or median (interquartile range). Abbreviations: AMI: acute myocardial infarction; LOS: length of stay.

## Data Availability

These data are publicly available with the Agency for Healthcare Research and Quality.

## Supplementary Materials

The following supporting information can be downloaded at: https://www.mdpi.com/article/10.3390/medicina58121846/s1, Table S1: Administrative codes used for identification of diagnoses and procedures; Table S2: Multivariable regression for in-hospital mortality in for LOS in AMI.

## Abbreviations

AMI	acute myocardial infarction
AOR	adjusted odds ratio
CABG	coronary artery bypass grafting
CI	confidence interval
CICU	cardiac intensive care unit
CS	cardiogenic shock
HCUP	Healthcare Cost and Utilization Project
ICD-9CM	International Classification of Diseases-9 Clinical Modification
IQR	interquartile range
LOS	length of stay
NIS	National/Nationwide Inpatient Sample
NSTEMI	non-ST-segment elevation myocardial infarction
OR	odds ratio
PCI	percutaneous coronary intervention
STEMI	ST-segment elevation myocardial infarction
