# Supplementary material for: Hospitalization Duration for Acute Myocardial Infarction: A Temporal Analysis of 18-Year United States Data
Source: Medicina (Kaunas). 2022 Dec 15;58(12):1846. doi: 10.3390/medicina58121846 (PMC9780977; doi:10.3390/medicina58121846)
Supplement: Supplementary file 1 [file medicina-58-01846-s001.zip › medicina-2088100-supplementary.pdf]

SUPPLEMENTARY TABLES

Supplementary Table S1. Administrative codes used for identification of diagnoses and procedures

Comorbidity	International Classification of Diseases 9.0 Clinical Modification codes
Cardiac arrest	427.5, 427.41, 99.60, 99.63
Ventricular tachycardia/fibrillation	427.4, 427.41, 427.42, 427.1
Coronary angiography	37.22, 37.23, 88.53-88.56
Percutaneous coronary intervention	00.66, 36.01, 36.02, 36.05, 36.06, 36.07, 88.57
Invasive hemodynamic assessment	89.63, 89.64, 89.66, 89.67, 89.68
Mechanical circulatory support	37.61, 37.68, 39.65
Invasive mechanical ventilation	96.7, 96.70, 96.71, 96.72
Hemodialysis	39.95
Hepatic failure	570.0, 572.2, 573.3, 573.4
Respiratory failure	518.81, 518.82, 518.85, 786.09, 799.1, 96.7, 96.70, 96.71, 96.72
Renal failure	584, 584.5, 584.6, 584.7, 584.8, 584.9
Hematologic failure	286.6-286.9, 287.4, 287.5
Neurological failure	293, 293.0, 293.1, 293.8, 293.81-293.84, 293.89, 293.9, 348.1, 348.3, 348.30, 348.81, 348.39, 780.01, 780.09, 89.14

Supplementary Table S2: Multivariable regression for in-hospital mortality in for LOS in AMI

Total cohort (N=11,622,528)		Odds ratio	95% confidence interval		*P*
			Lower Limit	Upper Limit	
**LOS ≤ 3 days**		Reference category			
**LOS > 3 days**		3.00	2.98	3.02	<0.001
**Age (years)**	**<75 years**	Reference category			
	**>75 years**	0.48	0.47	0.48	<0.001
**Female sex**		0.86	0.85	0.86	<0.001
**Race**	**White**	Reference category			
	**Black**	0.95	0.95	0.96	<0.001
	**Others[a]**	0.84	0.83	0.85	<0.001
**Primary payer**	**Medicare**	Reference category			
	**Medicaid**	1.07	1.05	1.08	<0.001
	**Private**	0.90	0.88	0.92	<0.001
	**Others [b]**	0.70	0.69	0.72	<0.001
**Year**	**2000-2005**	Reference Category			
	**2006-2011**	2.10	2.08	2.12	<0.001
	**2012-2014**	1.33	1.32	1.34	<0.001
**Quartile of median household income for zip code**	**0-25th**	Reference category			
	**26th-50th**	1.11	1.10	1.12	<0.001
	**51st-75th**	1.08	1.07	1.09	<0.001
	**75th-100th**	1.02	1.02	1.03	<0.001
**Hospital teaching status and location**	**Rural**	Reference category			
	**Urban Non-Teaching**	0.79	0.78	0.79	<0.001

	**Urban Teaching**	0.85	0.85	0.86	<0.001
**Hospital bed-size**	**Small**	Reference category			
	**Medium**	1.04	1.01	1.07	<0.001
	**Large**	1.08	1.05	1.11	<0.001
**Hospital region**	**Northeast**	Reference category			
	**Midwest**	1.26	1.25	1.28	<0.001
	**South**	1.18	1.17	1.19	<0.001
	**West**	1.29	1.28	1.30	<0.001
**Charlson Comorbidity Index**	**0-3**	Reference category			
	**4-6**	0.31	0.31	0.32	<0.001
	**≥ 7**	0.70	0.69	0.71	<0.001
**Type of AMI**	**ST-segment elevation**	Reference category			
	**Non-ST-segment elevation**	2.02	2.02	2.03	<0.001
**Multi-organ failure**		0.22	0.22	0.23	<0.001
**Cardiac arrest**		0.10	0.10	0.17	<0.001
**Cardiogenic shock**		0.39	0.38	0.39	<0.001
**Atrial fibrillation**		0.78	0.78	0.79	<0.001
**Atrial flutter**		1.13	1.11	1.15	<0.001
**Coronary angiography**		2.52	2.50	2.55	<0.001
**Percutaneous coronary intervention**		2.28	2.26	2.31	<0.001
**Coronary artery bypass grafting**		1.42	1.40	1.44	<0.001
**Pulmonary artery catheterization**		0.73	0.72	0.75	<0.001
**Mechanical circulatory support**		0.44	0.44	0.45	<0.001
**Non-invasive mechanical ventilation**		0.63	0.62	0.64	<0.001
**Invasive mechanical ventilation**		0.28	0.28	0.29	<0.001

**Acute hemodialysis**	0.47	0.46	0.48	<0.001

Legend: [a]Hispanic, Asian or Pacific Islander, Native American, Others; [b]Self-Pay, No Charge, Others

Abbreviations: AMI: acute myocardial infarction, LOS: length of stay
